# The endoluminal pressures during flexible gastrointestinal endoscopy

**DOI:** 10.1038/s41598-020-75075-9

**Published:** 2020-10-23

**Authors:** Yuki Ushimaru, Kiyokazu Nakajima, Masashi Hirota, Yasuaki Miyazaki, Kotaro Yamashita, Takuro Saito, Koji Tanaka, Tomoki Makino, Tsuyoshi Takahashi, Yukinori Kurokawa, Makoto Yamasaki, Masaki Mori, Yuichiro Doki

**Affiliations:** 1 Department of Next Generation Endoscopic Intervention (Project ENGINE), Osaka University Graduate School of Medicine, Center of Medical Innovation and Translational Research, Suite 0912, 2-2, Yamadaoka, Suita, Osaka 565-0871 Japan; 2 Department of Gastroenterological Surgery, Osaka University Graduate School of Medicine, Osaka, Japan; 3 Department of Gastroenterological Surgery, Toyonaka Municipal Hospital, Osaka, Japan; 4 Department of Surgery, Rinku General Medical Center, Osaka, Japan; 5 Department of Surgery and Science, Kyushu University Graduate School of Medicine, Fukuoka, Japan

**Keywords:** Gastrointestinal models, Oesophagus, Stomach

## Abstract

In flexible gastrointestinal (GI) endoscopy, endoscopic insufflation is crucial and directly affects visualization. Optimal visualization enables endoscopists to conduct better examinations and administer optimal treatments. However, endoscopic insufflation is typically performed manually and is subjective. We aimed to measure the GI endoluminal pressure during flexible GI endoscopy. Participants underwent esophagogastroduodenoscopy (EGD) at our endoscopy center. Pressure measurement was conducted after completing diagnostic or follow-up EGD. The endoluminal pressure in the esophagus and stomach was measured at 1-s intervals for 1 min while performing EGD for observational and diagnostic purposes. During the measurements, the endoscopists maintained what they subjectively considered to be adequate exposure for screening for lesions by dilating the lumen. Eighty patients were enrolled in this study. The upper GI endoluminal pressure was assessed during EGD without adverse events. The esophageal endoluminal pressure averaged 8.9 (− 3.0 to 20.7) mmHg, and the gastric endoluminal pressure averaged 10.0 (3.0–17.9) mmHg; the upper GI endoluminal pressures were not affected by patient-related factors or the number of endoscopists’ postgraduate years. We have successfully obtained the GI endoluminal pressures during EGD. Further accumulation of these data may lead to more stable and reproducible flexible endoscopic diagnosis and intervention.

## Introduction

In patients with digestive complaints, endoscopy is the gold standard for diagnosis, and it is often the primary examination technique used^1^. Upper gastrointestinal (GI) endoscopy (esophagogastroduodenoscopy, EGD) is a means of examination/treatment that is performed in daily clinical practice^2–8^. In examination and treatment with EGD, securing a working space is primarily based on obtaining adequate endoscopic exposure of the lining of the GI tract. Optimal visualization is established and maintained by insufflating the GI tract with an appropriate gas. For surgeons, it is routine practice to perform laparoscopic surgery under pressure control with automatic insufflation. We control the pressure appropriately to maintain the optimal visualization. However, endoscopic insufflation is currently performed under manual (subjective) control with no feedback concerning achieved pressure^9–11^.

Since the introduction of flexible GI endoscopy in clinical use, methods of insufflation have not been standardized, which sometimes results in problems associated with insufficient or excessive insufflation. Insufficient insufflation may lead to an incorrect diagnosis, such as missing a superficial cancer because of lack of definition of GI-wall stretch defects^12,13^. Additionally, in therapeutic endoscopy, insufficient insufflation may lead to incorrectly performing procedures, potentially causing adverse events (AEs) such as bleeding, perforation, and other dangerous consequences. Conversely, excessive insufflation may lead to serious AEs, such as Mallory–Weiss syndrome, Boerhaave syndrome (spontaneous rupture of the esophagus), and post-examination pain and bloating^14,15^. It has been assumed that skillful endoscopists subconsciously attain appropriate pressures for endoscopic procedures by manual insufflation. However, questions then arise as to how much pressure is needed and whether the optimal pressure depends on the target organ or the patient’s disease/condition. To the best of our knowledge, no published studies have investigated the appropriate pressure for endoscopic procedures. Further, to help standardize the insufflation procedure and thus facilitate more precise and reproducible diagnosis and safer and more appropriate interventions by all endoscopists, we believe that it is necessary to determine the GI endoluminal pressure during endoscopy.

Previously, we reported a new modality for endoscopic examination and intervention called “steady pressure automatically controlled endoscopy” (SPACE)^16–20^. We are convinced that SPACE can contribute to all types of diagnosis and treatment that are performed using flexible GI endoscopy. Currently, we are developing a dedicated endoscopic insufflation system for the clinical application of SPACE. For laparoscopic surgery, normal and low intra-abdominal insufflation pressure are defined as 12–15 mmHg and 5–7 mmHg for general cases, respectively^21^. However, for flexible GI endoscopy, there are no pressure data for more reproducible examination; data set accumulation is therefore important to be applied as a practical pressures for various procedures^22^. This pressure data can lead to endoscopic competency for novice endoscopists.

**Figure 1:**
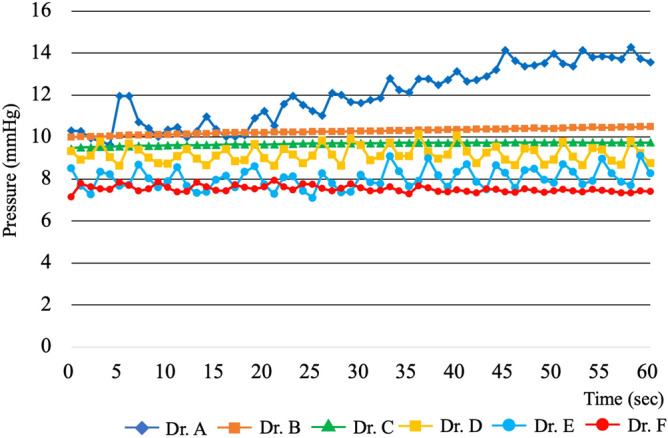
Time course of gastrointestinal (GI) endoluminal pressures during flexible GI endoscopy of the stomach in a single healthy volunteer. Endoluminal pressure during flexible GI endoscopy on a healthy volunteer performed in turn by six board-certified endoscopists was measured at 1-s intervals for 1 min. The endoscopists maintained the endoscopic exposure manually on a subjective judgement.

We believe that knowing the pressure in each hollow organ is essential for achieving ideal SPACE conditions, which are being currently worked on. As the pilot study, differences in GI endoluminal pressures achieved by different board-certified endoscopists performing what they considered to be “optimal” insufflation under the same condition and in the same individual were measured (Fig. 1). We found that assessment of “optimal” insufflation of the stomach is subjective, even among certified endoscopists, different endoscopists achieving different GI endoluminal pressures. This raised the question of whether guidelines for endoluminal pressure can be developed for a variety of subjects. Our study is the first clinical study aimed at measuring GI endoluminal pressure under various conditions. We included many patients, and endoscopic procedures were performed by physicians or surgeons with differing levels of experience. We collected pressure data attained during endoscopic examination.

## Results

### Patient characteristics

Eighty patients were enrolled in this study. Forty-nine patients out of them were conducted by expertise endoscopists (≥ 10 years). The other remaining 31 patients were conducted by novice endoscopists (< 10 years). The median age was 63 (range 27–82) years; 67.5% were men and 32.5% women. Seventeen patients had malignant neoplasms; twelve had early esophageal squamous cell carcinoma and six had early gastric adenocarcinoma (one had both types of carcinoma). Twenty-six (32.5%) patients had a history of endoscopic intervention, such as endoscopic submucosal dissection (ESD) and endoscopic mucosal resection (EMR). Thirty-six patients had hiatal hernias (including 33 accidental cases); 63 patients underwent EGD with antispasmodics: scopolamine butyl bromide, n = 38: glucagon, n = 16: and l-menthol, n = 9 (Table 1).

**Table 1:** Relevant patient characteristics.

Characteristic	Patients	Comments
Median age by gender, years (range)	Male, n = 54: 64 (35–82) Female, n = 26: 61 (27–79)	Total, n = 80: 63 (27–82)
Presence of malignant disease	Yes: 17 No: 63	Esophageal cancer (n = 12), gastric cancer (n = 6); one patient had both types of cancer
History of surgery	Yes: 26 No: 54	Esophageal cancer: CRT (n = 6), ESD (n = 2), EMR (n = 2) Gastric cancer: ESD (n = 12), partial gastrectomy (n = 4), EMR (n = 2); two patients underwent more than one procedure
Presence of hiatal hernia	Yes: 36 No: 44	Includes 33 contingent cases
Use of antispasmodics	Yes: 63 No: 17	Scopolamine butyl bromide (n = 38), glucagon (n = 16), l-menthol (n = 9)

Values are presented as number or median (range).

*CRT* chemoradiotherapy, *EMR* endoscopic mucosal resection, *ESD* endoscopic submucosal dissection.

### Endoscopic outcomes

**Figure 2:**
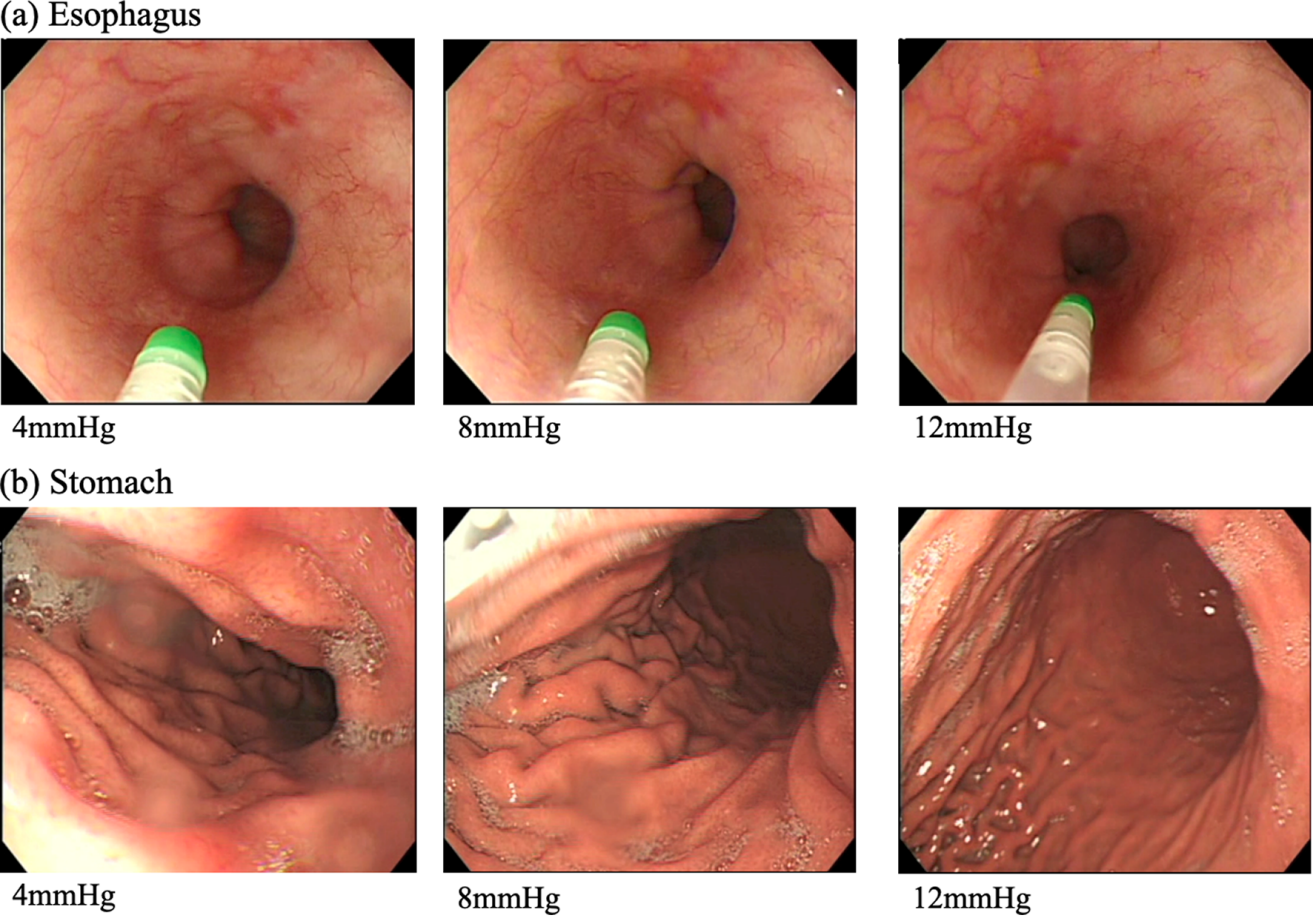
The correlation between endoscopic exposure and endoluminal pressure in esophagus and stomach. Endoscopic exposure of each endoluminal pressure condition in (**a**) esophagus and (**b**) stomach. The higher the endoluminal gastrointestinal pressure, the greater the dilation of the gastrointestinal tract.

**Figure 3:**
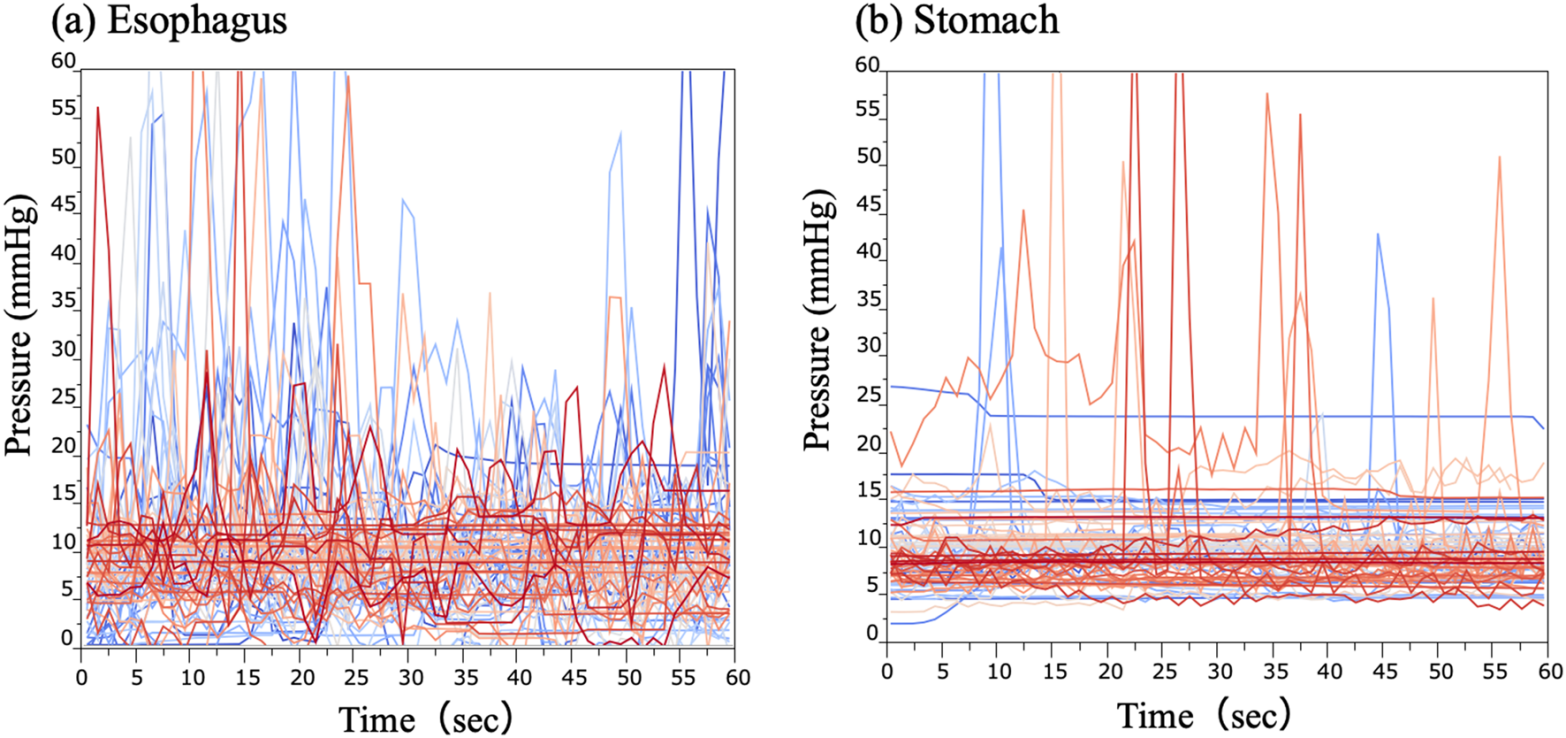
Changes in pressure in the esophagus and stomach during endoscopic examination. Endoluminal pressure transition in (**a**) esophagus and (**b**) stomach. Pressure fluctuations were larger and more frequent in the esophagus than in the stomach.

All endoscopic examinations were completed, including the acquisition of images of particular landmarks. Figure 2 shows an example of depict the correlation between endoscopic exposure and endoluminal pressure in one case (sampling data). Figure 3 shows endoluminal pressure transition in (a) esophagus and (b) stomach. After the endoscopic examination, upper GI endoluminal pressure measurements were also successful in all 80 patients. There were no endoscopic AEs such as bleeding, mucosal injury, or perforation.

### Pressure dynamics and characteristics

Figure 3 shows the endoluminal esophageal/gastric pressure dynamics and their characteristics. In the esophagus, the pressure fluctuated frequently (Fig. 3a); conversely, in the stomach, fewer fluctuations occurred and the fluctuations were smaller (Fig. 3b).

### Statistical analysis and endoluminal pressure profiling

**Figure 4:**
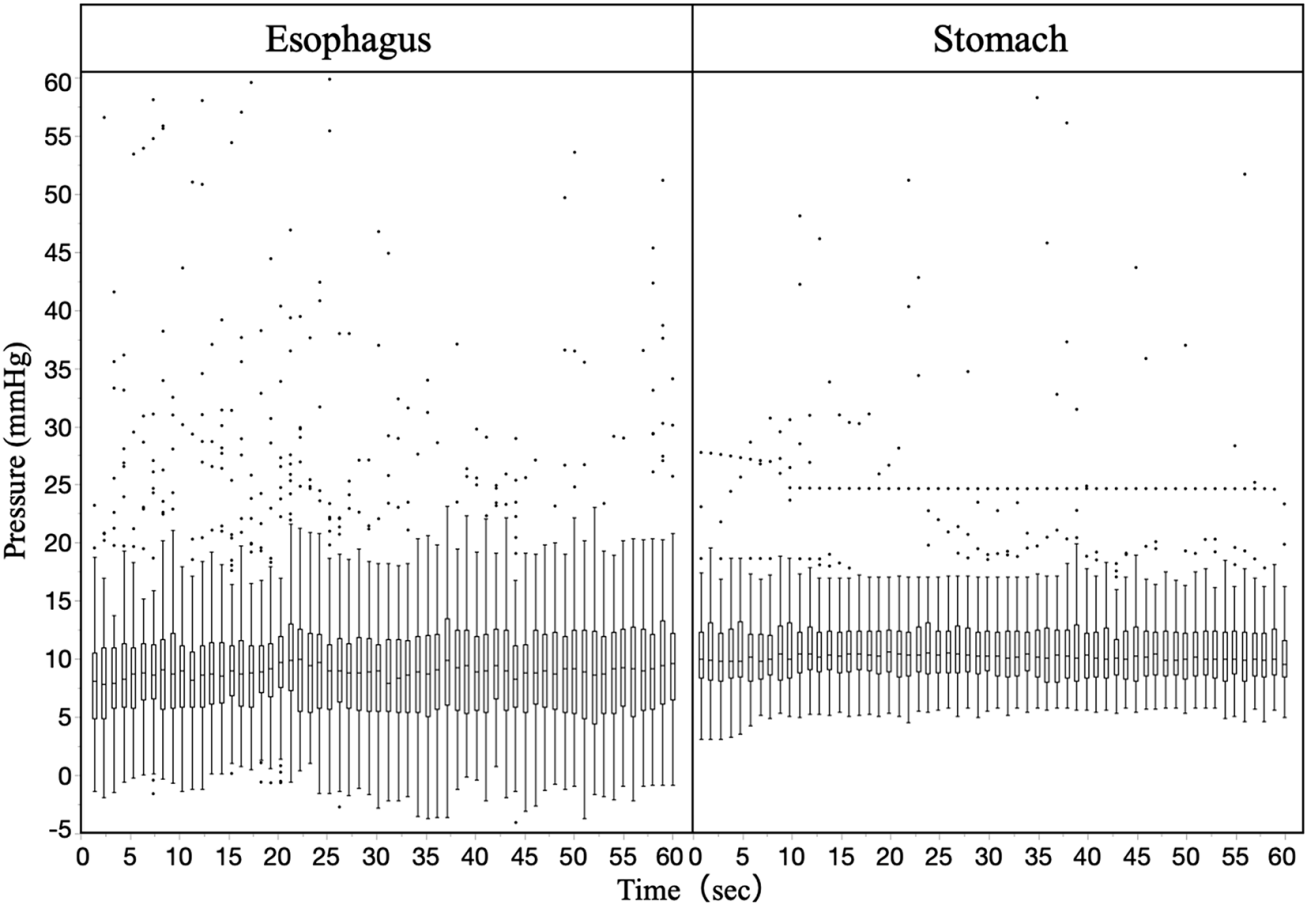
Time course of endoluminal gastrointestinal (GI) pressures achieved by manual insufflation during esophagogastroduodenoscopy. In 80 patients, the endoluminal pressures in the esophagus and stomach during flexible GI endoscopy were measured at 1-s intervals for 1 min. Endoluminal pressure fluctuations were larger and more frequent in the esophagus than in the stomach.

**Figure 5:**
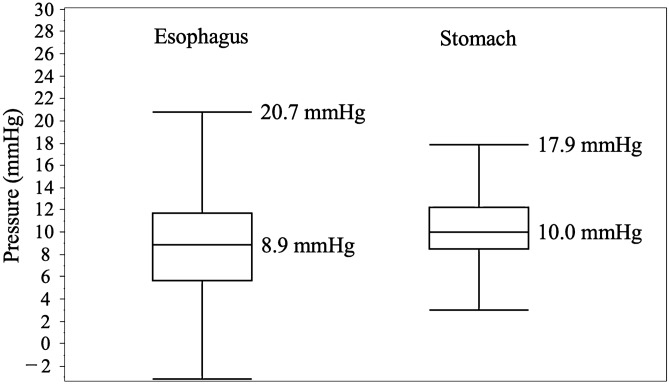
Median and range of endoluminal pressures achieved by manual insufflation during esophagogastroduodenoscopy (n = 80). The endoluminal pressure was lower in the esophagus than in the stomach, with the median endoluminal pressure being 8.9 mmHg (range − 3.2–20.7 mmHg) in the esophagus and 10.0 mmHg (range 3.0–17.8 mmHg) in the stomach.

**Figure 6:**
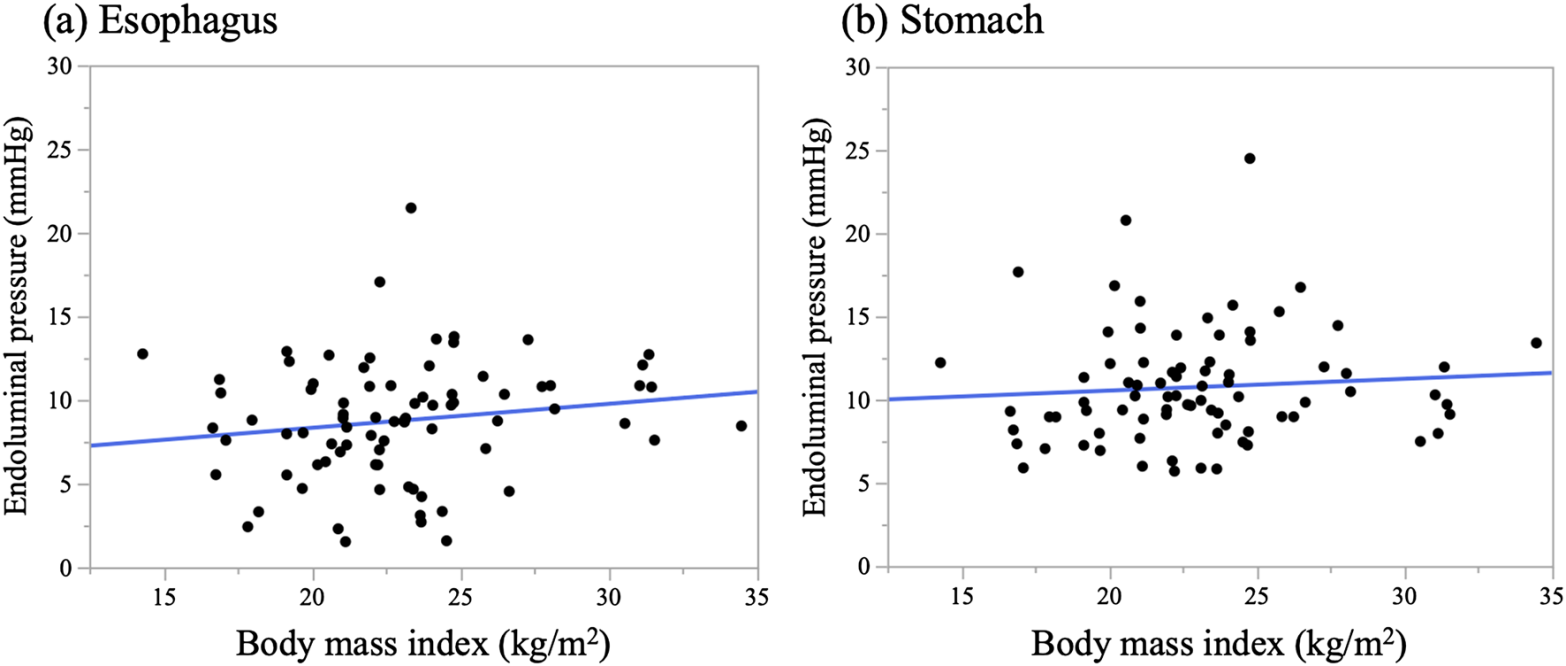
Relationship between body mass index (BMI) and endoluminal gastrointestinal pressure. The relationship between BMI and endoluminal pressure in (**a**) esophagus and (**b**) stomach. A positive correlation was found, but there was no significant difference.

The endoluminal pressures were statistically analyzed after classification at each time (s) and outliers were excluded. For visual clarity, box and whisker plots were created for each time point (Fig. 4). The median endoluminal pressure remained constant in both the esophagus and stomach. Figure 5 shows the box and whisker diagram for all pressure data regardless of time point. The endoluminal pressure was lower in the esophagus than in the stomach, with the median endoluminal pressure being 8.9 mmHg (range  − 3.2 to 20.7 mmHg) in the esophagus and 10.0 mmHg (range 3.0–17.8 mmHg) in the stomach. There were no statistically significant associations between median endoluminal pressure data and any of the background factors studied (Table 2). Figure 6 shows the relationship between body mass index and endoluminal GI pressure. A positive correlation was found, but there was no significant difference (Esophagus: R2: 0.0250, RMSE: 3.527, p = 0.161. Stomach: R2: 0.0066, RMSE: 3.416, p = 0.472).

**Table 2:** Association between patient characteristics and endoscopy pressure.

Characteristics	Details (n)	Esophagus, mmHg	*p*	Stomach, mmHg	*p*
Age, years	≥ 65 (44)	9.4 (1.5–21.5)	0.143	10.8 (5.7–20.8)	0.173
	< 65 (36)	8.3 (2.3–13.6)		9.8 (5.9–24.5)	
Gender	Male (54)	8.9 (1.5–21.4)	0.432	10.2 (5.8–24.5)	0.521
	Female (26)	8.6 (2.3–17.0)		9.9 (5.7–15.9)	
Body mass index, kg/m^2^	≥ 22 (49)	9.0 (1.6–21.5)	0.302	10.2 (5.7–24.5)	0.462
	< 22 (31)	8.3 (1.5–12.9)		9.4 (5.9–20.8)	
Presence of malignant disease	Yes (17)	9.0 (1.5–21.4)	0.449	10.8 (6.0–24.5)	0.277
	No (63)	8.6 (1.6–17.0)		9.8 (5.7–15.9)	
Presence of gastric cancer	Yes (7)	–	–	10.5 (6.0–24.5)	0.418
	No (73)			10.2 (5.7–17.7)	
Presence of esophageal cancer	Yes (13)	8.9 (1.5–12.7)	0.848	–	-
	No (67)	8.7 (1.6–21.5)			
History of cancer treatment	Yes (26)	10.0 (1.5–12.0)	0.546	11.3 (7.3–24.5)	0.094
	No (54)	8.7 (1.6–21.5)		9.7 (5.7–20.8)	
Presence of hiatal hernia	Yes (36)	8.8 (1.6–17.0)	0.387	9.8 (5.7–20.8)	0.389
	No (44)	8.8 (1.5–21.5)		10.6 (5.9–24.5)	
Use of antispasmodics	Yes (63)	8.7 (1.5–21.5)	0.095	10.2 (5.7–24.5)	0.832
	No (17)	10.2 (3.1–13.6)		9.8 (5.8–14.3)	
Examiner	Surgeon (40)	8.9 (2.4–13.8)	0.356	9.5 (5.9–20.8)	0.250
	Physician (40)	8.7 (1.5–21.5)		10.3 (5.7–24.5)	
Postgraduate years	≥ 10 (31)	8.7 (1.6–13.6)	0.441	10.3 (6.3–24.5)	0.158
	< 10 (49)	9.0 (1.5–21.5)		9.7 (5.7–20.8)	

Values are presented as number or median (range). *P* = 0.05 was considered to denote statistical significance.

## Discussion

The optimization of insufflation has always been a key to success in securing adequate visualization and working space for both endoscopy and laparoscopy^10,11,21,24–28^. Endoscopists and laparoscopists initially focused on the type of gas to use for insufflation. CO_2_ is superior to room air in terms of early absorption and clearance^29–33^, and it is therefore used globally for insufflation^21,34–36^. However, the insufflation procedure varies considerably between endoscopy and laparoscopy. Laparoscopic surgeons can easily set the insufflation pressure and receive feedback on the pressure achieved^35,36^, whereas endoscopists perform insufflation blindly without feedback on pressure. We thought that reproducible SPACE or pressure-regulated endoscopy might partially replace this manual insufflation; therefore, we believed that it was important to accumulate pressure data that was applied as a “appropriate value” or “benchmark” in various procedures^22^. Meanwhile, the question arose as to whether the endoluminal pressure at the time of endoscopy would be different depending on the organ, between the operators, and between the patients.

There have been no studies reporting on how GI endoluminal pressure varies during flexible endoscopy of the upper GI tract. Furthermore, there is no clear definition of “optimal” endoscopic insufflation. Thus, there may be differences in the GI endoluminal pressures obtained under the same conditions by "optimal" insufflation. This lack of information raised the question of whether guidelines for endoluminal pressure can be developed for a variety of subjects. We accordingly performed this study, in which we determined the median of endoluminal pressures in the esophagus and stomach during EGD and found there was a “frequently used” value of such pressures. We also found that the endoluminal pressure differed between the esophagus and stomach, which is the first reported finding, to the best of our knowledge.

In this study, we determined the median endoluminal pressures in 80 patients. Especially, the careful insufflation using appropriate pressure is necessitated when observing the EGJ opening as well as lower esophageal mucosal lesions, and when stretching the folds of the greater curvature of the stomach for searching the lesion. For these reasons, we designed this study by focusing on the esophagus and stomach. We considered that the endoluminal pressure achieved in the esophagus and stomach depended on various characteristics of those organs, such as shape, diameter, length, volume, and compliance. In particular, we considered that pressure was more stable in the stomach because of its relatively large capacity and consequent ability to function as a pressure reservoir. Conversely, the esophagus is elongated and would therefore have little capacity to buffer pressure, which results in more complex pressure profiles.

This compiled data series of endoluminal GI pressures will be the basic data for the clinical introduction of SPACE technology. In these studies, constant pressures were established and maintained in the GI tract with high reproducibility by using an automated insufflator while performing flexible GI endoscopy^16–20^. However, to date, there has been no study of the endoluminal GI pressure that endoscopists consider to be a gastrointestinal tract dilation suitable for endoscopic examination. The use of steady-pressure insufflation to achieve optimal pressures eliminates subjective biases and enables constant endoscopic exposure to be attained regardless of the endoscopist’s proficiency. This constant endoscopic pressure, in turn, contributes to stabilization of endoluminal pressures and standardization of endoscopic examinations and treatments. Improving the accuracy of endoscopic examination potentially improves its safety, reducing the burden on patients. Next-generation endoscopic treatment will require more complex and sensitive operations. We believe that SPACE technology with this pressure data can be expected to be useful and reliable for next-generation endoscopic treatment.

In the present study, the endoluminal pressures were obtained without AEs. This study did not include pressure data for healthy individuals because only patients with GI diseases were included. These diseases might have affected the original properties of the walls of the GI tract. Therefore, our data should not be considered as “normal” or “benchmark”. In this experiment, since the endoscopist was sufficiently skilled in performing EGD, the experiment was performed assuming that the performance was in sufficiently acceptable pressure values. Hence, the data obtained in this experiment were considered to reflect the generally sufficient endoluminal pressure. Figure 1 showed that there was intra-group bias in the “optimal” pressure for each endoscopist. From these results, pressure data of 80 cases were measured in order to obtain a reproducible index. Although Fig. 2 was just sample data, the endoscopic visualization was more optimized as the endoluminal pressure rise, till the pressure reaches to “plateau” level. Since skilled endoscopists recognize that there is no further improvement in visualization beyond the plateau pressure, they can practically avoid over-insufflation. By gathering more pressure data, more reliable and generalizable reproducible endoluminal pressure would be obtained. Such data can be applied as a reproducible pressure value in various procedures and used for maintaining the technical quality of procedures—as well as for learning, training, and credentialing purposes. Furthermore, linking of such a huge data set automatically to a hospital information system and incorporation of artificial intelligence would give rise to several new possibilities, such as “benchmark of endoscopic insufflation pressure” and “full-automatic endoscopic insufflation”, in the future.

This study has some limitations. First, the sample size was relatively small. Second, the study was conducted in a single facility and the participants were not healthy volunteers. Third, only a single type of endoscopic system was used; it is possible that pressure profiles differ between different brands of endoscopic systems. Fourth, in this study, we did not evaluate the endoscopic visualization of GI tract dilation during endoscopic examination. In the endoscopic examination, dilation of the digestive tract varies widely depending on the endoscopist. In addition, endoscopic insufflation is performed based on the subjective judgement of each endoscopist, and its quality and performance have not been objectively evaluated. We have not evaluated that point, and thus we should consider it in the next study. Fifth, endoluminal pressure was measured during diagnostic endoscopy, but not during therapeutic endoscopy. Moreover, given that therapeutic endoscopy is more dynamic than diagnostic endoscopy, the pressure profiles may be different. Sixth, the pressure data was measured independently of the endoscopic examination, and the measurement limited to 1-min measurements after scheduled EGD. Originally, we planned to measure the endoluminal pressures continuously during the entire EGD. However, since occupying a working channel with a pressure measuring probe might lead to problems such as insufficient suction, the inability to pass other forceps, disturbance of entire EGD procedures, etc. Therefore, we had to conduct pressure measurement sessions independently after routine EGD. Depending on the timing of measurement, the obtained pressure data might be different. Finally, we assessed only endoscope exposure and profiles of endoluminal pressure; whether such pressures are optimal regarding patients’ comfort is another matter. For patients, both the quality of endoscopic examination and treatment as well as minimization of post-endoscopic discomfort and AEs are important.

We succeeded in obtaining endoluminal pressures in the esophagus and stomach during endoscopic examinations. The use of these pressure data may lead to the standardization and uniformity of endoscopic procedures. The standardization of the insufflation procedure may lead to the determination of optimal GI endoluminal pressures for more precise diagnosis, as well as safer and more appropriate interventions by all endoscopists. For the novice endoscopists, the endoluminal pressure value may become the practical benchmark pressure in routine EGD. Next-generation endoscopy will be more complicated and challenging. Consequently, ensuring a reproducible pressure data for using SPACE or alternative technology will enhance those new procedures. Based on the data obtained from this study, we are finalizing the development of a pressure-regulated endoscopic insufflation system for clinical application.

## Methods

### Patients

This observational clinical study was registered with Osaka University Hospital. The participants underwent EGD at the Gastrointestinal Endoscopy Center of Osaka University Hospital (Department of Gastroenterological Surgery and Gastroenterology) from January 2011 through February 2013. The following data were obtained from prospectively registered endoscopic databases including: (1) patient demographics, (2) endoscopic outcomes (completion of endoscopic examinations, presence or absence of AEs), and (3) endoluminal pressure data profiles. The study was approved by the Osaka University Ethics Committee (clinical study registration numbers: 10219, 12049), and all research subjects gave written informed consent before study entry in accordance with the Declaration of Helsinki.

We included patients who: (1) were aged 20–80 years, (2) were undergoing examination of both their esophagus and stomach, and (3) gave written informed consent. The exclusion criteria included the presence of any of the following: (1) scirrhous-type gastric cancer with difficulty in obtaining adequate pressures by insufflation of the stomach; (2) advanced esophageal cancer causing obstruction; (3) history of any gastrectomy (distal, proximal, or total gastrectomy) for gastric cancer or any other gastric diseases; (4) history of esophagectomy for esophageal cancer or any esophageal diseases; (5) history of intestinal obstruction or current obstructive symptoms, such as severe abdominal pain with nausea or vomiting, according to the investigator’s judgment; (6) any life-threatening condition; and (7) otherwise judged inappropriate for inclusion by the investigating doctors. We included the partial gastrectomy cases without gastric deformity or stenosis, as minimal partial gastrectomies, especially for stromal tumor cases, did not affect gastric compliance and anatomical integrity.

### Endoscopic examination and manual insufflation

Thirty-one board-certified endoscopists conducted diagnostic or follow-up EGD; 17 surgeons and 14 gastroenterologists (postgraduate year; median 9 years, range 6–28 years). A high-vision flexible endoscope system (EVIS LUCERAGIF-260; Olympus, Tokyo, Japan) was used to perform all procedures with carbon dioxide. EGD was performed to observe following eight anatomical landmarks under optimal visualization; two points in the esophagus (proximal esophagus, Z-line), five in the stomach (cardia and fundus on retroflexed view; body, angulus on partial retroflexion; and antrum), and two in the duodenum (duodenal bulb, second part of duodenum)^23^. Pressure measurement was then conducted after completing those observation points. The endoscopist performed an endoscopy with manual insufflation to achieve what was subjectively considered an appropriate endoscopic exposure for lesion screening. During experiments, EGD was performed without using any sedatives.

### Endoluminal pressure measurements and endoscopic outcomes

Endoluminal pressure measurements was performed after scheduled follow-up EGD as it was without removing the endoscope. The measurements were taken in the middle thoracic esophagus and the gastric body. A spray catheter (Fine-jet catheter, W2816; TOP Corporation, Tokyo, Japan) was placed in the stomach via the instrument channel of the endoscope and connected to a digital manometer (MT-210F; Yokogawa Denki, Tokyo, Japan) to obtain GI endoluminal pressures, which were measured at 1-s intervals for 1 min.

The endoluminal pressure was measured while the endoscopists in turn maintained endoscopic exposure in the gastrointestinal lumen that they considered optimal for screening for lesions. In the pressure measurement, the endoscopists had no feedback on the measured endoluminal pressure. Subsequently, we analyzed the characteristics and pressure dynamics of the obtained data and statistically analyzed the measured pressure data. We then studied the relationships of GI endoluminal pressures with various background factors (patient age, gender, presence of malignant disease, treatment history, presence of hiatal hernia, use of antispasmodics, physician or surgeon examiner, and postgraduate year of the investigating doctor). Finally, any AEs were recorded.

### Statistical analysis

Statistical analyses were performed using a statistical software package (JMP, version 14.0.0; SAS, Cary, NC, USA). The results were presented as median (range). Student’s *t *test, the Mann–Whitney U test, or Pearson’s χ^2^ test were used to compare continuous and categorical variables, respectively. Categorical variables were also compared using Fisher’s exact test. Logistic regression analysis was used to investigate the relationship between the factors and the results. All values were two-tailed, and *p *values < 0.05 were considered to denote significant differences.

### Ethics statement

All procedures in this study were performed in accordance with the ethical standards of the responsible committee on institutional human experimentation and with the Helsinki Declaration of 1964 and later versions.
